# Interest in Insects: The Role of Entomology in Environmental Education

**DOI:** 10.3390/insects9010026

**Published:** 2018-02-23

**Authors:** Faith J. Weeks, Christian Y. Oseto

**Affiliations:** 1 Department of Biological Sciences, Towson University, 8000 York Road, Towson, MD 21252, USA; 2 Department of Entomology, Purdue University, 901 West State Street, West Lafayette, IN 47907, USA; osetoc@purdue.edu

**Keywords:** entomology education, arthropod education, invertebrate education, environmental education, student interest, teacher self-efficacy

## Abstract

University-based outreach programs have a long history of offering environmental education programs to local schools, but often these lessons are not evaluated for their impact on teachers and students. The impact of these outreach efforts can be influenced by many things, but the instructional delivery method can affect how students are exposed to new topics or how confident teachers feel about incorporating new concepts into the classroom. A study was conducted with a series of university entomology outreach programs using insects as a vehicle for teaching environmental education. These programs were used to assess differences between three of the most common university-based outreach delivery methods (Scientist in the Classroom, Teacher Training Workshops, and Online Curriculum) for their effect on student interest and teacher self-efficacy. Surveys administered to 20 fifth grade classrooms found that the delivery method might not be as important as simply getting insects into activities. This study found that the lessons had a significant impact on student interest in environmental and entomological topics, regardless of treatment. All students found the lessons to be more interesting, valuable, and important over the course of the year. Treatment also did not influence teacher self-efficacy, as it remained high for all teachers.

## 1. Introduction

Environmental education plays a vital role in helping students to understand not only the world around them but also their relationships to other living things. It is essential to remind individuals that the environment is an extension of themselves so its health is as important as their own [1]. While numerous environmental issues can be addressed locally, environmental education needs to address many of these concerns at the international level due to their subject matter, such as global climate change [2]. Environmental education is one way in which individuals all around the world can have a sufficient understanding of the natural world and environmental issues that will impact the future [3]. Formal education settings were found to be a promising way to use environmental education to promote environmental care, including positive attitudes, behaviors, and basic knowledge [4]. Environmental education has since become an important part of public school curricula, and research has shown that when environmental concepts are used to teach science, students held more positive beliefs and attitudes about the environment [5]. Previous research has found a positive correlation between student achievement and environmental education, although additional research to examine successful or innovative strategies for teaching environmental education is needed to determine how it supports student learning [6]. However, a key component to the successful integration of environmental education into the classroom is the teacher, and most teachers have not been educated in or trained to teach environmental concepts [7]. According to Mastrilli [8], environmental education needs to be integrated and consistently taught in the school curriculum before teachers feel comfortable with environmental topics. Teachers that do successfully link school curricula to environmental concepts, especially those found locally, help their students make connections between learning and the real world, which makes the information more concrete and meaningful [6]. When environmental education is not offered as a part of the curriculum, often outreach programs provide new methods of bringing environmental and entomological concepts and issues into the classroom.

According to the North American Association for Environmental Education [9], environmental education “teaches children and adults how to learn about and investigate their environment, and to make intelligent, informed decisions about how they can take care of it” (p. 1). Learning about animals and their role in the ecosystem is an important part of environmental education, although some animals, such as invertebrates, are not discussed due to negative human attitudes towards them. On this planet, invertebrates represent about 90% of all animal species, yet most people feel fear and a great dislike for them, especially insects and spiders [10]. According to entomologist E.O. Wilson [11], invertebrates are overall more important in maintaining ecosystems than vertebrates and if invertebrates were to disappear, the human race would be unable to survive more than a few months. Despite their great importance to almost every ecosystem on Earth, most people do not have a basic understanding of invertebrate life and are largely unaware of their importance [10]. Prior research has suggested that environmental education should use a variety of strategies at every possible opportunity to teach about invertebrates, including helping students focus on the many insects that are harmless and the great diversity of them in this world [12]. Studies with preservice elementary school teachers found that most will not teach about arthropods in their future classrooms even though this group of animals could assist science educators in teaching about ecosystem interactions [13]. Using insects as teaching tools is inexpensive, effective, and engaging for students, and it nurtures students’ natural curiosity about the world around them [14]. To further examine using entomology to teach environmental education, this study compared three common outreach delivery methods to determine their impact on student intrinsic motivation and teacher self-efficacy.

## 2. Theoretical Framework for the Study

### 2.1. Intrinsic Motivation

Intrinsic motivation is found when one engages in an activity for the pleasure and interest in it [15], and is a part of Self-Determination Theory. This theory postulates that events that increase an individual’s competence, or their feeling of being effective or confidence in a given situation, will enhance their intrinsic motivation [16]. Individuals are motivated to behave in a certain way for their own sake and not for reward or to avoid punishment, or because they are pressured by an external source [17], which will add to their intrinsic motivation. Intrinsic motivation will be reduced in situations in which the individual does not feel confident or because of a tangible reward [16]. In educational settings, motivation is absolutely necessary for effective instruction, as it has been positively correlated with student achievement, such as effort and grades [18]. By getting students excited about course content, this excitement can lead to students enjoying their learning, which can lead to students focusing on the process of learning rather than just on their grades or the approval of others [19]. Therefore, one research objective of this study was to determine if students have a higher interest in environmental and entomological topics and issues when (1) taught by an entomologist, (2) taught by teachers trained by an entomologist, or (3) taught by teachers with no entomological training. This study focused only on intrinsic motivation in the hope that student interest in environmental and entomological concepts would continue in their lives outside of structured lessons and the classroom.

### 2.2. Teacher Self-Efficacy

This study examined participating teachers’ self-efficacy in the classroom. Self-efficacy is defined as ‘the individual’s perceived expectancy of obtaining valued outcomes through personal effort’ [20] (p. 7), which is grounded in Social Cognitive Theory [21]. An individual’s performance at a task is influenced by their self-efficacy, and it can change based on how the individual rates the results of that performance. When applied to the classroom, teacher self-efficacy is the instructor’s belief in his/her ability to organize and deliver those things necessary to accomplish a particular teaching task in a specific context [22]. Teacher self-efficacy is influenced by the prior experiences of the teacher, including their successes, failures, and feedback from others [23]. Bandura [24] stated that “teachers’ beliefs in their personal efficacy to motivate and promote learning affects the types of learning environments they create and the level of academic progress their students achieve’’ (p. 117). Research examining teachers with high teacher self-efficacy have found numerous positive characteristics that are necessary for student learning. These teachers are more open to new ideas and teaching methods that could better meet the needs of their students [25], and they are more likely to put more effort into problem solving [26]. Hence, our study sought to determine if teachers have a higher teacher self-efficacy when (1) trained by an entomologist, (2) passively observing an entomologist, or (3) they have no contact with an entomologist.

## 3. Literature Review

To examine student intrinsic motivation and teacher self-efficacy, this study chose to compare different ways in which environmental education is provided by universities to the community. Numerous universities have environmental education outreach programs, ranging in delivery methods from one-time classroom presentations to large assembly programs. Three of the most common methods are Scientist in the Classroom, Teacher Training Workshops, and Online Curriculum. Scientist in the Classroom programs are selected by the classroom teacher, inviting an expert into the classroom to teach a specific subject. This delivery method allows elementary school students to interact with researchers in specific fields, providing the students with role models that may influence future career choices [27,28]. The literature on visiting scientist programs shows that the instructors act as mentors to students, providing them with new knowledge and demonstrating the importance of science in the world [27]. These programs provide additional resources that classroom teachers often lack and information that teachers may not feel comfortable teaching due to their limited expertise [29,30,31].

Teacher Training Workshops are typically one-day events in which experts provide teachers with new curricula, materials, and knowledge focused on one topic. Teacher Training Workshops are considered to be an effective way of sharing new research and curricula with multiple teachers over a short amount of time [31,32]. To effectively motivate change and learning, it is important to have teachers take the role of the student in such workshops [33], and modelling effective teaching strategies has been suggested as a way to enhance teacher self-efficacy [34]. Teachers’ perceptions about the usefulness of the training will result in their decision to attend, seek other sources, or to not include the topic in their curriculum [31]. This decision could influence what types of science students are exposed to, excluding those topics that cannot be effectively taught as outreach programs.

Online Curriculum is a method in which experts write and post lesson plans for classroom use, and the classroom teacher must take the initiative to learn and obtain the materials necessary to convey the information. Providing local teachers with new curriculum is a simple way of delivering new ideas, science, and activities into the classroom because few materials are needed by outreach providers. According to Davis and Krajcik [35], there are many factors that influence the effectiveness of a curriculum.

Specifically, teachers’ use of and learning from text-based curriculum materials depend not only on the characteristics of the curriculum materials but also on the type of teaching activity in which the teacher is engaged, the teacher’s own knowledge and beliefs…how those beliefs are aligned with the goals of the curriculum, and the teacher’s disposition toward reflective practice (p. 4).

Luehmann and Markowitz [31] found that partnerships between universities and schools gave teachers access to these resources, which teachers claimed were important to their curriculum development and students’ access to science.

## 4. Materials and Methods

### 4.1. Study Participants

To recruit for this study, all schools that taught the fifth grade in the 19 counties surrounding the research institution’s county in Indiana, USA, but had not participated in any entomological outreach events were randomly assigned by random number generator to one of the three delivery methods. All fifth grade teachers were asked to voluntarily participate. Of the 105 schools contacted, eight public elementary schools chose to join, resulting in twenty classrooms, fifteen teachers, and 518 students in the study (Table 1). The fifth grade was selected due to its ability to use environmental and entomological topics to meet state science standards. Teachers ranged in age from 28 to 57 years old, with students ranging in age from 9 to 12 years old. All teachers identified themselves as white for ethnicity, and the student population was predominately white as well (78%–98%). The number of students that received free or reduced lunch ranged from 32 to 63%. All teachers reported receiving no entomological training prior to the study. All subjects or their guardians gave their informed consent to participate in this study after its protocol was approved by the Institutional Review Board of Purdue University.

### 4.2. Study Design

To answer our research questions, four lesson plans using insects as the vehicle to teach environmental education were created to meet fifth grade state science standards. These lessons were based on the 5E instructional model [36] and were written to be taught individually or combined into a thematic unit with connecting themes and an optional unifying activity. For this study, the lessons were treated as a thematic unit, including the optional unifying activity, and taught over the course of one school year with at least one month separating all lessons and assessments.

The first lesson focused on the definition of an ecosystem, the characteristics all insects share, different insect mouth types, and how an insect’s specific ecosystem and mouth types influence their food choices. The second lesson discussed the role of insects in an ecosystem according to their food choices on the trophic pyramid. Insect decomposers and their contributions to an ecosystem were explored in lesson three, and lesson four focused on insect predator/prey relationships and the importance of balancing the trophic levels in an ecosystem. All lessons used live arthropods in activities native to the students’ state, including bess beetles, painted lady butterflies, ladybugs, mealworms, house crickets, giant water bugs, carpet beetles, termites, millipedes, woodlice, darkling beetles, dragonfly nymphs, carpenter ants, tobacco hornworms, milkweed bugs, and praying mantids. The only non-native insect used in a lesson was the Madagascar Hissing Cockroach. The optional activity occurred at the end of each lesson, with a unifying activity at the end of the environmental education unit. This activity used picture representations of all the live insects used during the unit and discussed the results of removing specific predators or prey from their ecosystem.

Due to time constraints in the schools, participants were assessed using a pretest-posttest design in which testing was separated by two lessons, resulting in three testing periods (base test, mid test, final test). The base test was administered at least one month prior to teaching the thematic unit, the mid test was administered during the unit between lessons 2 and 3, and the final test was given approximately one month after the conclusion of the unit.

The Scientist in the Classroom treatment consisted of an entomology professor presenting the four lessons to each classroom, in his own teaching style, using the provided presentation materials and activities. The selected professor, Dr. Tom Turpin, has been a leading expert in entomology education for the past 30 years and has won nearly every teaching award in entomology education, as well as teaching awards from professional societies. The Teacher Training Workshop delivery method consisted of the same entomology professor from the first treatment conducting two events—one in the fall for the first two lesson plans, and another in the spring for the last two lesson plans. These workshops modeled the lessons for teachers, and included background information for each lesson plan. Teachers were provided with all materials to conduct the activities and taught each of the lesson plans in their classroom, in their own teaching style. Finally, the Online Curriculum treatment included teachers accessing the four lesson plans online then teaching each lesson, in their own style, to their classroom with no interaction with the entomologist. Teachers in this treatment were provided with all materials to conduct the activities at the same times as the other two treatments, with the first two lessons posted in Fall and the last two lessons posted in Spring. A researcher was present at all lessons in each treatment to determine if all key elements were taught and to deliver live insects.

### 4.3. Data Collection and Analysis

#### 4.3.1. Intrinsic Motivation

To determine the impact of using entomology to teach environmental education on student interest in this area, Deci and Ryan’s [37] Intrinsic Motivation Inventory (IMI) was administered to all student participants due to its connections to Self-Determination Theory. This assessment is a multi-scaled instrument used to examine the way in which participants relate to a particular activity and their subjective reaction to their experience. Four of the seven subscales were selected for this study (Interest/Enjoyment, Effort/Importance, Value/Usefulness, and Pressure/Tension) due to their relevance to the research questions, totaling 27 questions answered using a 7-point Likert scale. The Interest/Enjoyment subscale focuses solely on assessing intrinsic motivation while the Effort/Importance subscale seeks to show relevance. The Value/Usefulness subscale examines the internalization of the activities, and the Pressure/Tension subscale is a negative predictor of intrinsic motivation. These subscales were modified to focus on the environmental and entomological topics discussed in the four lesson plans, as Bandura [21] recommended creating assessments of self-efficacy specific to the task being analyzed (Table 2).

Student IMI scales were scored according to Deci and Ryan [37] scoring procedures, then averaged by class for each subscale. To compare the three treatments, a mixed model Analysis of Variance (ANOVA) was conducted using the Statistical Packages for the Social Sciences (SPSS) with time as a random factor, treatment as a fixed factor, and the subscale class average as the dependent variable. For each classroom, the time variable identified the subscale class average score as either the base test score, mid test score, or the final test score. Tukey post-hoc tests were conducted to determine differences between the treatments, and Cohen’s *d* effect sizes were calculated. To ascertain the overall impact of the lesson plans on student intrinsic motivation, paired t-tests and Cohen’s *d* effect sizes were calculated with the class averages.

#### 4.3.2. Teacher Self-Efficacy

Teacher self-efficacy when teaching environmental education with insects, through the lens of Bandura’s Social Cognitive Theory, was assessed using The Teachers’ Sense of Efficacy Scale by Tschannen-Moran and Woolfolk Hoy [25]. This measurement consists of 24 questions on a 9-point Likert scale, separated into three subscales: Efficacy in Student Engagement (ESE), Efficacy in Instructional Strategies (EIS), and Efficacy in Classroom Management (ECM). These questions were modified to focus teacher responses on their beliefs about teaching an environmental education thematic unit, their feelings about teaching environmental education and entomology, and their confidence in controlling the classroom when using such activities (Table 3). Teacher assessments were also administered at the base test, mid test, and final test. Due to the extensive experience and expertise of the professor, he did not complete any assessments during this study. Table 4 provides an overview of the study, including its objectives, theories, and these assessments for both teachers and students.

The Teachers’ Sense of Efficacy Scale was scored according to Tschannen-Moran and Woolfolk Hoy [25] for the three subscales. The three treatments were analyzed using Cohen’s *d* effect sizes to determine any differences between the treatments. Paired t-tests and Cohen’s *d* effect sizes were used to determine the overall effect of the four lesson plans on teacher self-efficacy when using entomology to teach environmental education.

### 4.4. Study Limitations

While over 500 fifth grade students participated in this study, the number of elementary teachers was far lower. This small sample size may have affected the results of this study, leading to a limitation in the generalizability of its discussion. To compensate for this, Cohen’s *d* effect sizes were calculated for differences between the treatments, as this analysis method emphasizes the size of the difference between groups by standardizing the difference between two means [38]. The effect size measures how many standard deviations the experimental group is above the average participant in the control group, which examines effectiveness of the treatment without conflating effect size and sample size [38].

## 5. Results

### 5.1. Student Intrinsic Motivation

Analysis of student interest data comparing their base test scores to mid test scores found no significant differences between treatments for all four subscales, but all students showed significant increases in their responses for the Interest/Enjoyment, Value/Usefulness, and Effort/Importance scales (Table 5). For the Interest/Enjoyment scale, effect sizes indicate that the Scientist in the Classroom treatment scores did not increase as much as the Teacher Training Workshops (*d* = 0.64) or Online Curriculum (*d* = 0.84) treatments. Effect sizes for the Value/Usefulness subscale also revealed this trend, with medium effect sizes found when comparing the Scientist in the Classroom treatment to the Teacher Training Workshops (*d* = 0.34) and Online Curriculum (*d* = 0.51) treatments. Analysis for the Effort/Importance subscale indicated a large effect size in favor of the Online Curriculum treatment, demonstrating that students in this condition put in more effort and placed more importance on lessons one and two than students in the Scientist in the Classroom (*d* = 0.94) and Teacher Training Workshops (*d* = 1.36) treatments. For the Pressure/Tension subscale, large effect sizes indicate that students in the Online Curriculum treatment felt more pressure or tension than those in the Scientist in the Classroom (*d* = 1.26) and Teacher Training Workshops (*d* = 1.42) conditions.

Comparing student interest results from their mid test to the final test also revealed no significant differences between the three treatments, but significant overall increases were found for the Interest/Enjoyment, Value/Usefulness, and Effort/Importance subscales, as well as a significant decrease for the Pressure/Tension subscale (Table 5). Analysis of the Interest/Enjoyment subscale found a medium effect size when comparing the Online Curriculum treatment to the Scientist in the Classroom (*d* = 0.41) and Teacher Training Workshops (*d* = 0.70) treatments. This trend was also found in the Value/Usefulness and Effort/Importance subscales, with students in the Online Curriculum treatment indicating that they chose value/usefulness and effort/importance more often than those in the Scientist in the Classroom (*d* = 0.60, *d* = 0.84) and Teacher Training Workshops (*d* = 1.18, *d* = 1.52) conditions for lessons three and four. Minimal effect sizes were found for the Pressure/Tension subscale when comparing mid test to final test student interest scores.

When comparing the base test to the final test, analysis revealed no significant differences between treatments, yet significant increases by all students for the Interest/Enjoyment, Value/Usefulness, and Effort/Importance subscales (Table 5). Analysis also found that students in the Online Curriculum treatment found all lessons more interesting and enjoyable than students in the Scientist in the Classroom (*d* = 0.79) and Teacher Training Workshops (*d* = 0.62) conditions. Students in this condition also showed increases over the four lessons than those in the Scientist in the Classroom (*d* = 0.60, *d* = 0.94) and Teacher Training Workshops (*d* = 0.97, *d* = 1.36) treatments for the Value/Usefulness and Effort/Importance subscales, respectively. Again, students in the Online Curriculum treatment indicated more pressure and tension than the Scientist in the Classroom (*d* = 0.40) and Teacher Training Workshops (*d* = 0.80) treatments.

### 5.2. Teacher Self-Efficacy

Teachers in the Online Curriculum treatment expressed higher teacher self-efficacy for the ESE and EIS subscales when compared to teachers in the Scientist in the Classroom (*d* = 0.76, *d* = 0.62) and Teacher Training Workshops (*d* = 0.69, *d* = 0.44) treatments. For the ESE and ECM subscales, large effect sizes were found when comparing the Online Curriculum treatment to the Scientist in the Classroom (*d* = 1.16, *d* = 0.99) and Teacher Training Workshops (*d* = 0.70, *d* = 0.95) treatments. Analysis of the subscales revealed large effect sizes for the ECM subscale where teachers in the Online Curriculum treatment indicated feeling less self-efficacious in their classroom management than the Scientist in the Classroom (*d* = 0.78) and Teacher Training Workshops (*d* = 0.86) treatments. All results regarding teaching self-efficacy should be regarded as provisional evidence of differences, as all but one of the statistically significant effects can be deemed not practically significant.

## 6. Discussion and Implications

### 6.1. Student Intrinsic Motivation

This study provides evidence that university-based environmental education outreach can have an impact on student interest in various topics, in this case using entomology to incorporate environmental education in the science curriculum. While no differences were found when comparing the three outreach delivery methods, student scores did significantly increase overall, indicating that their intrinsic motivation towards the environmental and entomological unit increased with each lesson. This finding suggests that the specific outreach method may not be necessarily as important as just getting entomology and live arthropods into classrooms. Entomology can be used to help capture student interest and show them the value and importance of environmental education, although additional research is necessary to further examine the impact of these delivery methods. Schools that are unable to afford inviting an entomologist to their classroom or to send their teachers to an all-day professional development workshop can still incorporate successful environmental education outreach activities into their curriculum through online lesson plans created by experts across many contexts and subjects. These lesson plans, regardless of the way in which the topics are presented to the students, can meet teachers’ need for state standards while injecting new environmental topics into the classroom, as well as provide students with more information about ecosystems in the local area. This research suggests that universities currently offering environmental education outreach programming may wish to establish a strong online presence in addition to their in-person outreach offerings.

Analysis of the subscales found that students in both the Teacher Training Workshops and Online Curriculum treatments reported more interest and value in lessons one and two than students in the Scientist in the Classroom treatment. This result may be because these two treatments were delivered by a teacher with more training and experience teaching fifth grade students, and students may have responded favorably to the teacher that could meet their specific learning needs. Outreach programs that utilize teachers trained in teaching specific age groups may enhance student knowledge of the topics, but more research is needed to determine how to match a teacher to the needs of the audience [39]. Students in the Online Curriculum condition put more effort in and placed more importance on these two lessons than students in the other treatments, which may be due to the lack of contact their teachers had with the entomologist. These teachers needed to put in more energy to teach these lessons, as they could not rely on the entomologist to teach the lesson or model the lesson for them [27]. Students may have viewed this instruction as more like normal classroom teaching than something novel, whereas the students in the Scientist in the Classroom and Teacher Training Workshops may have viewed the lessons as more novel and separate from typical classroom learning. As for the pressure/tension subscale, the teachers in the Online Curriculum treatment may not have known how to properly handle or work with the live insects, as they were not shown by an expert nor had any training in entomology prior to this study. As a result, their students may have expressed more concern about using live animals in the activities, as fear and disgust are common reactions to seeing an invertebrate [10,12]. An important part of teaching entomology is getting the audience comfortable with the insects, and working with an entomologist or having one teach the lesson are ways in which to alleviate the tension felt when working with these animals. Including videos or explicit instructions on how to handle different insects, as well as more information about their specific habits, may help teachers using online lesson plans to feel more comfortable utilizing invertebrates in their classroom and to assure their students that the animals will not harm them.

For lessons three and four, the students in the Online Curriculum condition found more interest, value, and importance in the lessons than students in the other two treatments. The two teachers in this treatment may have put more energy into modifying these lessons for their students, as the activities and concepts were more complicated than lessons one and two. Concerns about their students understanding the material and working with the larger live specimens may have motivated them to adapt the lessons, as both of these teachers indicated to the researcher that they tried to connect the material to prior lessons, outdoor experiences, and students’ everyday lives. These slight changes may have encouraged more student engagement in the material, resulting in an increase in their intrinsic motivation [18,19]. Lesson plans on the internet may want to encourage teachers to alter the lesson to meet their needs and may offer suggestions on how to do so from teachers that have successfully taught the lesson in their own classroom. Future studies on how to mold online lesson plans to different students’ needs are important, as more teaching materials are being offered via the internet.

When comparing the treatments across the entire study, students in the Teacher Training Workshops and Scientist in the Classroom treatments felt less pressure and tension from the lessons than students in the Online Curriculum condition, which again could be due to a lack of teacher comfort and/or experience in training, holding, or working with live insect specimens. Insects are not discussed in most classrooms because of a negative attitude, a lack of training, and the teacher’s background experience with insects [14]. Besides teaching educators about invertebrates and how to utilize them in the classroom, students may benefit from a brief introduction to the specific insects used in that lesson to help alleviate any negative feelings they may have, including how to properly hold them and use them in the activities. Students in the Online Curriculum treatment, however, responded positively on the other three subscales of the IMI resulting in higher rankings of effort, value, and interest for all four lessons. Again, these scores may be a product of the teachers need to put more effort into learning and teaching the lessons, which they may have modified to better meet the needs of their students and their own teaching style. Future research may wish to examine the relationship between Online Curriculum student motivation and their reports of higher pressure and tension, as these feelings may negatively influence student intrinsic motivation. Additional research will be needed to examine this finding in more depth, including qualitative interviews and analyzing the materials these teachers created for each environmental education lesson.

### 6.2. Teacher Self-Efficacy

Analysis of the teacher assessments suggests that teachers with high teacher self-efficacy will remain at those levels when new environmental and entomological lessons are included in their science curriculum, regardless of the outreach delivery method. Previous research has found in-service teacher self-efficacy to be difficult to change and sustain, and experienced teachers appear to have stable teacher self-efficacy beliefs even when exposed to new teaching methods and professional development [22]. However, there is also the possibility that these teacher self-efficacy scores are artificially high due to an overestimation of teachers’ beliefs of what they should be reporting rather than their own actual teacher self-efficacy beliefs [5]. These teachers may also be reporting unrealistic optimism about their teaching by rating themselves above average, as many teachers will only select the higher values when assessed for their teacher self-efficacy [40]. Additional research is needed to determine the impact of these delivery methods on teachers with low teacher self-efficacy, if the inclusion of entomology when teaching environmental education influenced their feelings about teaching the science unit, and different teacher self-efficacy measures to triangulate teacher responses with their actual beliefs when teaching the lessons. However, these results show that the use of live insect specimens and entomological topics did not lower teacher self-efficacy beliefs, which suggests that the use of insects as tools may be an effective way of teaching environmental and science topics. This study is limited by its reliance on self-reporting teacher self-efficacy measures, and the possibility that using a different scientist may affect the results. More research into teacher reasoning for their rankings and beliefs about their teaching would benefit our understanding of this field.

Analysis of subscale data demonstrates that those teachers that have no contact with an entomologist feel less self-efficacious about their classroom management in this teaching environment as the lesson progresses. This result may be because lessons three and four contained more difficult material and activities that built on simpler concepts covered in lessons one and two, or this change may be from the inclusion of larger live insect specimens, including the praying mantid and dragonfly larvae. Teachers in the Online Curriculum condition also had no additional support to teach these lessons, such as seeing the lesson modelled in the Teacher Training Workshops treatment or being able to rely on an expert in the Science in the Classroom treatment, which may result in these teachers feeling less control over how their students will react to the lessons or supervising students handling the live animals. Online outreach programs may wish to include more support for teachers that need additional help, such as supplementary materials, tips from other teachers on conducting activities, and videos of educators modelling the lesson. These aids may help teachers feel more self-efficacious and in control of their classrooms if they see the lesson prior to teaching it or get feedback from other in-service teachers. These conditions may also explain the drop in self-efficacy for these teachers regarding student engagement in lessons three and four, especially if these teachers were less sure of how their students would respond to the larger insect specimens or more complicated activities included in these lessons. Posting videos of the lessons, background information about the insects, or models of how to properly hold such animals may be needed to alleviate teacher concerns about student engagement in these activities.

Teachers working in the Online Curriculum treatment may also have been more willing to join a condition where they had no contact with an entomologist because of their high and stable teacher self-efficacy beliefs, which may account for their higher rankings at the beginning of the study for student engagement and instructional strategies. These teachers may have felt surer of their ability to take the information and materials provided and modify them to meet the needs of their students, as they were not offered any additional support. The two teachers in this treatment did adapt the lessons more to their teaching styles than those in the other treatments, which also may contribute to their higher feelings of teacher self-efficacy. While a larger sample size will be necessary to confirm these findings, this research suggests that more support may be needed with environmental education lessons including more complicated concepts or less understood animals, like insects, to maintain that high teacher self-efficacy needed to promote student learning. More research is needed to investigate why these teachers chose to participate in such a condition, their reasons behind modifying the lessons as they did, and why other participating teachers chose not to modify the lessons as much. The small sample size is the largest limitation of this study, as teachers are often difficult to recruit into research for a variety of reasons [33], and additional research will be necessary to confirm both student and teacher findings.

## 7. Conclusions

This research demonstrates the important role of environmental education outreach in the classroom, as well as the success of using entomology to teach environmental concepts. Whether teaching two lesson plans (to the mid test) or four lesson plans (to the final test), these outreach programs increased student interest and enjoyment in the topic, the effort and importance they placed on the subject matter, and the value and usefulness they found in that material. These programs can expose students to new environmental topics, how they relate to each other, and their importance in nature, which may influence the value these students place on the environment in the future. Teacher self-efficacy did not decrease over this study, suggesting that using local insects is a hands-on, engaging, cost-effective method for teaching about relationships in an ecosystem. While these results are specific to the use of entomology for teaching environmental education, this research can apply to other environmental fields that may benefit from examining their own outreach education programs, as well as encourage other university departments to offer these types of programs. Research such as this is still needed to examine possible long lasting impacts on student interest in environmental and entomological education, and its impact on the teacher self-efficacy of classroom educators, as well as on the evaluation of other outreach delivery methods that may assist in educating the public about important environmental and entomological issues.

## Figures and Tables

**Table 1:** Demographic information for student and teacher participants in each treatment.

	Scientist in the Classroom	Teacher Training Workshops	Online Curriculum
Schools	3	3	2
Classrooms	7	8	5
Teachers	6	7	2
Male	1	1	1
Female	5	6	1
Students	187	208	123
Male	99	97	62
Female	88	111	61

**Table 2:** Sample questions from the IMI [37], modified to include environmental and entomological themes.

Subscale	Example Questions
Interest/Enjoyment	I enjoyed doing the activities with insects very much. I would describe the activities with insects as very interesting.
Effort/Importance	I did not put much energy into the activities with insects. It was important to me to do well at the activities with insects.
Value/Usefulness	I think that doing the activities with insects are useful for understanding different insect roles in an ecosystem. I would be willing to do the activities with insects again because it has some value to me.
Pressure/Tension	I was very relaxed in doing the activities with insects. I felt pressured while doing the activities with insects.

**Table 3:** Sample questions from The Teachers’ Sense of Efficacy Scale [25], modified for the environmental and entomological lessons.

Subscale	Example Questions
Efficacy in Student Engagement (ESE)	How much can you do to help your students value learning about entomology? How much can you do to improve the understanding of a student who is failing during the entomology lessons?
Efficacy in Instructional Strategies (EIS)	How well can you respond to difficult questions from your students when teaching about and with insects? How much can you do to adjust your insect lessons to the proper level for individual students?
Efficacy in Classroom Management (ECM)	How much can you do to get children to follow classroom rules during insect lessons? How well can you respond to defiant students when you are teaching about and with insects?

**Table 4:** Overview of the study for both students and teachers.

	Research Questions		Delivery Method *	Theoretical Framework	Assessment
Students	Do students have a higher interest in environmental and entomological topics and issues when:	Taught by an entomologist	SC	Self Determination Theory (Deci and Ryan)	Intrinsic Motivation Inventory [37]
		Taught by teachers trained by an entomologist	TTW		
		Taught by teachers with no entomological training	OC		
Teachers	Do teachers have a higher teacher self-efficacy when:	Trained by an entomologist	TTW	Social Cognitive Theory (Bandura)	The Teachers’ Sense of Efficacy Scale [25]
		Passively observing an entomologist	SC		
		Having no contact with an entomologist	OC		

* SC = Scientist in the Classroom; TTW = Teacher Training Workshop; OC = Online Curriculum.

**Table 5:** Mean differences for the four subscales of the IMI [37] for all students, regardless of treatment.

	Mid-Base Test	Final-Mid Test	Final-Base Test
Interest/Enjoyment	0.50 **	0.19 **	0.69 **
Value/Usefulness	0.58 **	0.23 **	0.81 *
Effort/Importance	0.48 **	0.23 **	0.71 **
Pressure/Tension	−0.04	−0.12 *	−0.16

* *p* < 0.05; ** *p* < 0.001.
